# Understanding In Vitro Tissue Culture-Induced Variation Phenomenon in Microspore System

**DOI:** 10.3390/ijms22147546

**Published:** 2021-07-14

**Authors:** Piotr Tomasz Bednarek, Katarzyna Anna Pachota, Wioletta Monika Dynkowska, Joanna Machczyńska, Renata Orłowska

**Affiliations:** Plant Breeding and Acclimatization Institute—National Research Institute, Radzików, 05-870 Błonie, Poland; katarzyna.anna.pachota@gmail.com (K.A.P.); w.dynkowska@ihar.edu.pl (W.M.D.); j.machczynska@ihar.edu.pl (J.M.); r.orlowska@ihar.edu.pl (R.O.)

**Keywords:** plant tissue cultures, signaling pathways, transcriptome functioning, compounds methylation, biochemical cycles, DNA mutations

## Abstract

In vitro tissue culture plant regeneration is a complicated process that requires stressful conditions affecting the cell functioning at multiple levels, including signaling pathways, transcriptome functioning, the interaction between cellular organelles (retro-, anterograde), compounds methylation, biochemical cycles, and DNA mutations. Unfortunately, the network linking all these aspects is not well understood, and the available knowledge is not systemized. Moreover, some aspects of the phenomenon are poorly studied. The present review attempts to present a broad range of aspects involved in the tissue culture-induced variation and hopefully would stimulate further investigations allowing a better understanding of the phenomenon and the cell functioning.

## 1. Introduction

Tissue culture-induced variation (TCIV) is a well-established phenomenon of any changes affecting regenerants phenotype or genotype during in vitro plant regeneration [1,2,3,4]. If the changes are stably inherited via the generative cycle, it is usually called somaclonal variation (SV) [5,6]. However, the TCIV and SV terms are often used mutually [7]. TCIV may be pronounced at the morphological [8], biochemical [9], genetic [10] and epigenetic [11] levels, which do not necessarily overlap [12]. Possibly the most investigated level is based on DNA markers.

The studies of molecular aspects of TCIV started at the beginning of the 1980s [13,14,15]. The development of the restriction fragment length polymorphism (RFLP) [16] and the randomly amplified polymorphic DNA (RAPD) [17] techniques (Table 1) resulted in a burst of experimental data suggesting that even using anther culture, plant regeneration was subjected to numerous mutations such as single nucleotide polymorphisms (SNPs) [18]. Some studies suggested that variation was due to the so-called pre-existing variation [19,20], the others addressed mutations to the activation of transposable elements [21] due to DNA methylation pattern alternations [22]. For a long time, the origin of changes was not apparent. It was speculated that they might have come from the degradation of the cells during tissue cultures [23], the elevated production of reactive oxygen species species (ROS) [24], due to stressful conditions [25]. The degradation hypothesis claimed that cell death in culture under stress and programmed cell death leads to secondary metabolites (pigments, tannins). Their release into culture can induce somaclonal variation in an unspecified way [23]. It was speculated that the callus phase and indirect embryogenesis could have contributed to the phenomenon [26]. For about a decade, it was thought that studies on TCIV were deficient. However, the development of new molecular tools and progress in epigenetics led to the revision of previous data and pushed a deeper understanding of the phenomenon.

The introduction of the amplified fragment length polymorphism (AFLP) approach [27], followed by the methylation-sensitive amplified polymorphism (MSAP) one [28,29], allowed studies of DNA methylation changes addressed to in vitro tissue culture plant regeneration [30,31]. Although that changes are frequent, but may vary in wide spectrum, from 0.07% [32] to 52% [33]. While the MSAP approach was productive, it could identify only DNA methylation changes related to CG and CHG sequence contexts [34]. Moreover, the ways to quantify changes used distinct algorithms [35] that might have differed from study to study [36,37], making a comparison of results somewhat tricky. Thus, the semi-quantitative MSAP approach, allowing for quantifying DNA methylation changes, was suggested to overcome the limitation [38]. However, studies on sequence variation required utilization of additional marker techniques. Consequently, another AFLP based technique, the so-called methylation sensitive AFLP (metAFLP) approach, was developed [1,39], allowing quantification of sequence and DNA methylation changes during a single experiment. Further advances in the technique showed that one might study not only CG and CHG but also the CHH methylation context, an essential tool in studies on cereals [1,12].

The development of the new generation sequencing (NGS) opened innovative opportunities. The diversity arrays technology methylation analysis (DArTseqMet) techniques permitted for identifying a large number of markers that could be employed in quantification procedures evaluated for the semiquantitative MSAP approach [40]. MethylRAD [41] or MethylSeq [42] are the other alternatives. The MethylSeq method is an NGS variant of the bisulfite-based sequencing approach. It could be used to study different types of genomic DNA methylation, but its application requires at least 10X coverage. The MethylRAD approach uses Mrr-like enzymes to collect 32-bp methylated DNA fragments from the whole genome for high-throughput sequencing. It allows for de novo methylation analysis using low DNA input. The two approaches have incredible potential in DNA methylome studies; however, they were not yet used in tissue culture studies.

All the techniques mentioned above were exploited in studies on the whole genome. When it was of value to investigate DNA methylation pattern change of a specific sequence, the bisulfite approach was recommended [43,44]. It allowed the establishment of the extent to which the CG, CHG, and CHH sequences were methylated in the genome [45]. Thus, the tools to study molecular aspects of the TCIV were established. Moreover, by that time, the genetic model of the studies was also evaluated [46]. It became apparent that studies on TCIV should start from a well-defined, preferentially homozygous double haploid plant that served as a donor of tissue for the regenerants subjected to further analysis. Utilizing specific plant materials and sophisticated molecular marker techniques, TCIV was studied in barley [47], triticale [48], *Polyscias filicifolia* [49] and *Gentiana pannonica* Scop. [4]. It was shown that the phenomenon is common to plants [50,51,52] and that at least some DNA methylation changes could be transmitted to the progeny [53,54]. Furthermore, DNA methylation changes affecting regenerants may require several generative cycles to be stabilized [48]. Using a modification of the metAFLP approach that utilized primers directed towards TEs, their putative role in sequence variation was demonstrated [47,55]. However, the mechanisms of the TCIV are still being discussed.

It is becoming understood that analyses of DNA methylation changes affecting tissue culture due to plant regeneration are insufficient to have an image of the phenomenon. Interdisciplinary studies are needed to have species-specific models describing consecutive steps required to change the cell fate towards somatic development. In this context, studies of the cell wall, cell membrane, subintinal layer, their components, and the way signals transmitting stresses are involved in the process are needed. An interesting approach to study the role of the cell wall or biochemical pathways affected by in vitro tissue culture is attenuated total reflectance Fourier transform infrared (ATR-FTIR) spectroscopy [56,57], which allows for the identification of putative cell wall components [58] or biochemical pathways [59] participating in sensing stresses. It was documented that β-glucans being built of glucose units [60], and probably present in the subintinal layer of some microspores [61], may be sources of glucose for new anther-culture-derived regenerants [62]. Furthermore, stressful conditions might influence DNA methylation, probably disturbing the methionine cycle [62]. The role of copper and silver ions in sequence variation [63] and green plant development [40] was also documented in barley regenerants derived via anther culture. Similarly, in embryo-derived regenerants of barley copper ions participated in sequence variation, possibly via modification of methylated sequences [12]. The respective relationships could be evaluated using mediation and moderation analyses [64] applied to DNA methylation changes and the ATR-FTIR spectroscopy data. Such results could be used in structural equation modeling (SEM), resulting in a deeper insight into the phenomenon leading towards its practical applications. However, due to limited sample size, SEM analysis allowing construction of an in vitro plant regeneration either via andro- or embryogenesis was not evaluated. If not the most substantial, a further limitation is the understanding of the role of the way the cell senses and transmits stresses leading towards TCIV.

The review aims to present data related to the in vitro tissue culture-induced variation, how stressful conditions may affect tissue culture, the role of the cell wall, cell membrane, mucilage layer, signaling compounds, and biochemical pathways.

### 1.1. Stresses and Their Role in Plant Regeneration through Tissue Culture In Vitro

It is assumed that inductive stress treatment is necessary to initiate the cell reprogramming process required for the microspore to switch from gametophytic to embryogenic fate. The stress treatment needed to switch the developmental fate of microspores depends on the plant species and the species genotype. Osmotic, starvation, cold, heat, and many other stresses are employed to initiate andro- and embryogenesis [67,68] (Table 2).

Three phases of embryogenic development due to androgenesis could be distinguished. The first one reflects the acquisition of embryogenic potential as the reaction to stress. At this stage, which lasts about three days, repression of gametophytic development and dedifferentiation of the cells occurs. The second stage follows the induction and culture initiation step. The microspores divide and produce multicellular structures persisting within the microspore wall (exine) [74], indicating the initiation of embryogenesis. This stage, at least in barley, may take two days. Next, the exine breaks down, and embryos follow a similar zygotic embryogenesis pathway through globular, heart, torpedo, and cotyledonary embryos. The formation of tissue culture structures reflects the third phase of embryogenic development that begins around the 21st day of in vitro tissue cultures [74]. Interestingly, the third phase of embryogenic development corresponds to the point when under increased copper concentration present in induction medium, regeneration of new barley plants via anther culture results in the highest output [40].

In the model system of *Hordeum vulgare* (barley), the inductive cold stress (4 °C) is applied, whereas, in *Brassica napus*, 32 °C is used to initiate a switch from gameto- to sporophytic path. Stress-related heat-shock proteins (HSP70 and HSP90) were detected in microspore embryogenesis cultures of *Brassica napus* and *Capsicum annum* due to cold treatment [75]. Thus, a protective role for the chaperones was suggested; however, their role in microspore embryogenesis is not clear. Low temperatures may alter endogenous ethylene (ET) levels enhancing tolerance with higher ET concentrations [76].

During reprogramming stages, induced stresses result in increased cell death and oxidative stress. At this point, ROS arise in excess and are accumulated [77]. However, in the case of *Medicago sativa* leaf protoplasts, the application of oxidative stress-inducing agents resulted in acceleration of cell cycle re-entry that was accompanied by a lower level of ROS accumulation [78]. The equilibrium between ROS-scavenging and ROS-producing mechanisms administrates the cell’s level of damage and oxidative stress. Several enzymes of the antioxidative machinery of the cell increase their activity in microspore cultures. These stress-related proteins may have a protective role [79].

Abiotic stress factors may impact auxin homeostasis [80] probably resulting in the induction of somatic embryogenesis [81,82,83]. While microspore culture does not require exogenous auxin (2,4-dichlorophenoxyacetic acid), endogenously, the phytohormone may participate in microspore reprogramming in *Brassica napus* and in vitro embryo formation [84]. It was shown that induction of microspore embryogenesis resulted in de novo endogenous auxin biosynthesis and accumulation of indole-3-acetic acid (responsible for embryo patterning, polarization, and differentiation) in pre-embryo cells starting from the first embryogenic divisions [84]. Auxin activity, biosynthesis, and transport are essential for stress-induced microspore embryogenesis. There is a link between auxin biosynthesis, its perception, transport, gene expression, signaling, and nontranscriptional responses; however, the exact mechanisms remain elusive [85,86,87]. The other hormone vital for plant cells is cytokinin that acts antagonistically to auxin. Cytokinin participates in cell growth and may control the early stages of somatic embryogenesis [88]. Spatiotemporal localization of cytokinin and auxin responses during microspore embryogenesis was suggested [74]. However, data concerning the presence of endogenous cytokinin in microspore embryogenesis was not evidenced.

It is worth mentioning that stress conditions may induce or impact autophagy, a significant pathway for recycling cell materials [89,90]. Autophagy may promote plant cell survival under starvation and stress conditions [91]. Moreover, excretion of cytoplasmic material (occasionally containing whole organelles) via single membrane-bound autophagic bodies and those deposited in the cell wall (remnants from the digestion of cytoplasmic organelles) of embryogenic microspores in between the cell wall of embryogenic microspores and the plasma membrane was observed [92]. Some of the autophagosomes were transported out of the cell, creating extracytoplasmic fibrillar and membranous material deposits It was shown that embryogenic microspores are associated with autophagy and excretion of the removed material [92]. Thus, autophagy is a kind of cytoplasmic cleaning, whereas excretion is a mechanism of avoiding unnecessary vacuolar system growth [92]. The excretion was essential for proper microspore embryogenesis; however, in some cases, the material was redirected to the cell [92], possibly implying ‘recycling’ of the materials for cellular processes. It should also be mentioned that autophagy can involve converting the lytic vacuole to a storage one [93,94].

The connection between ROS and autophagy in plants was suggested [95]. In barley, the activation of autophagy after inductive stress at 4 °C with upregulation of *HvATG5* and *HvATG6* genes, and a rise in the number of autophagosomes was reported [96]. Autophagy is activated and involved in cell death with the participation of cathepsins the proteases that degrade proteins during stress-induced microspore embryogenesis in barley [96]. It was also demonstrated that stress treatment of *Brassica napus* also resulted in induction of autophagy [92] in parallel to cysteine-dependent proteases (metacaspase) involved in programmed cell death, stress and cell proliferation [97], and gene expression [98]. Interestingly, application of agents directed towards ROS (MnCl_2_), autophagy [3-methyladenine (3-MA), inhibitor], and protease activities, caspase 3-like, and metacaspase activities (E64, Ac-DEVD-CHO, and Ac-VRPR-FMK; inhibitors), reduces cell death levels, increasing embryogenesis in rapeseed and barley [96] opening up pathways reducing stress-induced cell death at the early stages of microspore embryogenesis.

Inductive stresses are responsible for cell reprogramming. The process involves DNA demethylation and de novo methylation [99]. A global DNA hypomethylation during the change of the gametophytic to sporophytic fate and first embryogenic divisions were observed in barley [100]. Among inductive stressful conditions, darkness is one of the most exploited in cereals [101]. When mannitol is applied, triggering microspores to dividing [73], the culture is subjected to osmotic, and carbon starvation discussed earlier [102]. Sometimes, instead of darkness, heat stress is applied [103]. It was suggested that ethylene participates in the response of plants to heat stress [76]. Stresses induce ethylene accumulation. The higher the ethylene concentration, the higher the chance of plant survival under stressful conditions [104]. Most probably, ethylene modulates gene expression via ethylene signaling mechanisms [105].

The tissue culture medium itself may be treated as a stressful factor. The regeneration medium contains many ingredients such as 6-benzylaminopurine, 2,4-dichlorophenoxyacetic acid, alpha-naphthalene acetic acid, and indole-3-acetic acid not necessary are neutral to the cells may induce TCIV [101,106,107]. As such ingredients may be toxic, they may lead to genomic DNA degradation as indicated by comet analysis [101]. It was also demonstrated that copper and silver ions containing media might also affect DNA methylation patterns [12], resulting in DNA sequence changes [62]. Moreover, the two ions most probably change respiratory chain balance affecting the Yang cycle [62] and inducing sequence changes [63]. Interestingly, copper and silver may moderate relationships between de novo methylation/demethylation processes, leading to green plant regeneration [40]. For more details on that, see an excellent review published recently [74].

Assuming that different stresses may affect in vitro tissue culture, the mucilage layer, the cell wall, and the plasma membrane are reasonable candidates for sensing stresses [108,109] affecting the cell functioning and possibly promoting the TCIV. However, the information on how the mucilage layer, the cell wall, and plasma membrane perform due to in vitro tissue culture plant regeneration is not well addressed.

### 1.2. The Mucilage Layer

The mucilage layer is formed on the surface of cells. It consists of polysaccharides, proteins including histones, etc., and extracellular DNA (exDNA), known as danger-associated molecular patterns [110,111,112,113]. The components of the layer may activate the innate immune system. ExDNA is defined as “DNA located outside the cell and originating from intracellular DNA by active or passive extrusion mechanisms or by cell lysis” [114]. It is assumed that it participates in immune defense in plants and contributes to “damage-associated molecular patterns” (DAMPs). Depending on DNA origin (self-exDNA (endogenous) or non-self-exDNA (exogeneous)) may act differently. Non-self-exDNA in plants results in reactive oxygen species formation and callose deposition [115]. On the other hand, self-exDNA may inhibit root growth, the effect not identified in non-self-exDNA. It was also demonstrated that self-exDNA acts in a concentration-dependent manner [116,117,118] by reacting with pattern recognition receptors [119]. Its action depends on the self-damage level recognized by plants. The presence of methylated or non-methylated CpG sequences seems important in plant reaction and recognition of endo- and exogenous DNAs [120]. It was also demonstrated that self-exDNA participates in immune signaling [117], stimulating calcium signaling and plasma membrane decomposition in *Zea mays* [117]. Furthermore, endogenous DNA may inhibit seed germination and trigger H_2_O_2_ production following by mitogen-activation protein kinase (MAPK) in *Phaseolus vulgaris* [118]. The MAPK signaling pathway senses extracellular signals and transmitting them from the cell membrane to the nucleus in response to various environmental stimuli [121]. Still, the mechanism of exDNA action is not understood. Most shreds of evidence on exDNA role come from studies on plant roots [122,123]. The importance of protein constituents of the mucilage layer and its treatment with proteases was suggested [124]. It was also shown that DNase I may lead to necrosis under pathogen inoculation of root tips [125]. Still, the role of the mucilage layer decomposition and secretion of DNA molecules from surrounding cells remains uncovered.

Even less is known in the case of microspores. Microspores are cells surrounded by a primary and secondary wall with haploid nucleus specifically surrounded by mitochondria present in the vicinity of the nucleus’s pores. It was demonstrated that mitochondria might cover microspore surface and vegetative nuclei around postmeiotic mitosis I (a few days before flowering). The mitochondria were also detected apart nucleus (shortly after postmeiotic mitosis) in 30 nm from a nuclear pore [126]. It should be stressed that, under dark conditions, a callose subintinal layer is formed [127]. The presence of callose might be linked to microspores’ ability to switch from gameto- to sporophytic fate [128]. Furthermore, the autophagy phenomenon responsible for the secretion of damaged organelles outside the cell was observed in stressed microspores [96]. These phenomena are in line with the mucilage layer’s presence in the case of microspores. The layer may sense some abiotic stresses, inducing an innate immune response and allowing or enhancing the possibility of anther-culture-derived plant regeneration. Most probably, the layer may interact with the cell wall and or cell membrane.

### 1.3. Cell Wall and Plasma Membrane as Sensors of Stresses

Even less, the pollen cell wall protects male sperm from abiotic stresses (i.e., abrasion, desiccation, and UV radiation). A durable wall consists of gametophyte-derived intine and sporophyte-derived exine layers covered with lipid-rich pollen coat or trephine [129,130]. The intine resembles the primary cellulosic plant cell wall [129,131,132] whereas the exine is formed of heterogenous polymer composed of polyhydroxylated aliphatic constituents with aromatic or conjugated side chains containing ether and ester bonds [133,134,135] called sporopollenin synthesized from precursors in tapetum [129,130] and anchored on to microspores [136].

Pollen wall formation starts after microspore meiosis generating tetrads covered with callose. At the late tetrad stage, the microspore surface is formed of primexine, a transient cell wall matrix-shaped of polysaccharides outside of microspore plasma membrane inside callose. Primexine is composed of sporopollenin. This layer may contain cellulase [137]. The callose wall degrades after the tetrad phase allowing free microspore release into the locule and tecta. At this stage, a thin microspore-derived intine wall forming between the developing exine and a microspore-derived intine wall is evidenced [138].

As the composition of the microspore cell wall is not apparent, available information on the plant cell wall in general with emphasis on cellulose and wall matrix polysaccharides [139] are discussed. All of them form a rigid structure. The essential cell wall function relies on providing mechanical strength to resist turgor pressure. It forms a barrier against biotic and abiotic stresses that may alter the cell wall components’ deposition [140].

The cell wall cellulose may be present in amorphic, semi amorphic, or crystalline forms. Crystalline cellulose has significantly better stiffness than all other constituents. Amorphous cellulose is more penetrable and accessible to enzymes and has a higher enzyme binding capacity than its crystalline counterpart so that it has a higher hydrolysis rate. The ratio of these two regions characterizes crystallinity. The cellulose with high crystallinity usually has low enzymatic hydrolysis efficiency [141,142,143]. Cellulose synthase (CESA) complexes (CSCs) synthesize cellulose microfibrils assembled in the Golgi apparatus. CSCs actively synthesize cellulose when delivered to the plasma membrane (PM). CSCs move along cortical microtubule paths that define cellulose microfibrils synthesis. CSC traffic between the PM and various intracellular compartments plays a vital role in determining the level of cellulose synthesized [144]. Uridine diphosphate glucose (Glc) originates from the cytosolic invertase/uridine diphosphate Glc phosphorylase pathway [145]. It is the substrate for CESAs [146]. The CESA proteins are involved in the dimerization/oligomerization of CESA subunits [147] due to a cytosolic N-terminal region. Moreover, the proteins consist of two transmembrane domains, a large cytoplasmic central loop that contains the substrate binding and catalytic regions, six additional transmembrane domains [148,149], and an intracellular C-terminal domain. The CSC rosettes contain 18 CESA subunits synthetizing 18 glucans [150].

Phytohormones may influence cell wall composition. For example, the amount of cellulose increases in methyl jasmonate, whereas lignin content decreases after salicylic acid application. The profile of gene expression involved in cell wall biosynthesis is also modified [151]. Phytohormone signaling pathways regulate stress perception at the primary cell wall, followed by cellulose synthesis and microtubule arrangement [140]. Alternatively, ROS and peroxidases arising in response to stresses in the cell wall may cross-link phenolic compounds and glycoproteins, resulting in stiffening. If ROS-levels remain high during continued stress, OH°-radicals lead to polymer cleavage [139]. Cellulosomes could accomplish the same, an extracellular supramolecular multienzyme complex that can efficiently degrade cellulose and hemicelluloses in plant cell walls [152].

The role of cellulose in sensing stresses was demonstrated on mutant plants. Cellulose-deficient mutants are typically more sensitive to abiotic stress than wild-type plants [140]. Thus, the cellulose synthesis machinery may be vital in abiotic stress responses [140]. The other option is that some cell wall components may sense stresses via cell wall integrity [153]. The cell wall proteins, i.e., the *Arabidopsis thaliana* leucine-rich repeat receptor kinase LRR-RK male discoverer 1-interacting receptor-like kinase 2 (MIK2) was suggested to play such a role [154]. The MIK2 regulates cell wall damage responses upon cellulose biosynthesis inhibition due to abiotic stress [153]. MIK2 has overlapping functions with receptor-like protein kinase THESEUS 1 (THE1), also proposed as a cell wall integrity sensor. Moreover, plants may coordinate stress responses by integrating phytohormones (auxins, cytokinins, gibberellins, abscisic acid, ethylene, brassinosteroids, salicylic acid, jasmonates, and strigolactones) and their pathways [140,155,156].

Recent studies have demonstrated that cellulose forms participate in a signaling pathway that links cellulose and mitochondria. Two *cell wall maintainer1* (*cwm1*) and *cwm2* pentatricopeptide repeat protein genes were shown to be involved in editing mitochondrial transcripts encoding subunits of respiratory complexes (i.e., complex III linked to the maintenance of cell wall integrity under stress) and activation of retrograde mitochondrial signaling via ANAC017, a transcription factor participating in retrograde signaling to the nucleus upon mitochondrial dysfunction [157,158]. A complex hierarchy of transcription factors exists downstream of ANAC017. These involve ANAC and WRKY transcription factors associated with organellar signaling and senescence; moreover, the network includes ethylene- and gibberellic acid-related transcription factors involved in stress responses [159].

The development of plants requires the biosynthesis, deposition, and degradation of cell wall matrix polysaccharides. Biosynthesis of polysaccharides is performed in the endoplasmic reticulum and Golgi of the plant secretory system by polysaccharide glycosyltransferases (GTs) transmembrane proteins. The synthesis of the cell wall matrix glycans (pectins, xylans, xyloglucans, mannans, mixed-linkage glucans (MLGs), and arabinogalactan components of arabinogalactan proteins (AGPs) proteoglycans) was mapped to multigene families [160]. Different stresses can perturb targeted genes to promote functional adaptation [161]. The polysaccharide fraction of the cell wall may participate in stress sensing [162]. The environmental (mechanical) signals affect the cell wall determining its phenotypic features. However, the way stress is sensed and transmitted into the cell is still under investigation.

The plasma membrane was suggested to participate in signal transduction because of its position at the interface between the cell’s interior and the cell wall. Thus, the crucial players that maintain cell status and respond to stresses are in the plasma membrane’s same vicinity [163]. Interestingly, the layer between the plasma membrane and cell wall, the so-called subintinal layer present in microspores with embryogenic fate and composed of callose, is also considered essential in stress sensing. As callose is formed of glucans, it may serve as a source of glucose [164] required by glycolysis which impacts the Krebs cycle and the electron transport chain.

Cereal β-glucans have a specific combination of β-(1→4) and β-(1→3) linkages into linear long-chain polysaccharides of high molecular weight. β-glucans were identified in barley and oats [165]. Under elongated dark conditions, β-glucans deliver glucose for glycolysis, supporting biosynthesis of, i.e., lipids [166] that may act as signaling compounds [167,168], affecting gene expression [169,170]. Thus, the subintinal layer components are reasonable candidates for signals [171] of stresses in microspore culture. It was also demonstrated that β-glucans might impact DNA methylation of the CHG context in barley [62]. Whatever is the sensor of abiotic stresses at the cell wall level, the signal needs to be transmitted to the cell. It is being accepted that calcium is a second messenger that facilitates responses to stresses by activating calcium-binding proteins [172,173,174]. Another candidate that can sense stress and may act as a cell wall mechanism protecting plants from external stresses is β-1,3-linked D-glucose [140]. Furthermore, evidence shows that massively glycosylated hydroxyproline-rich proteins called AGP improve plant regeneration in barley anther culture [175]. The AGP content changes in *Brassica napus* are related to the developmental fate of microspores [176]. The proteins are present in the mucilage layer, the cell wall, and the cell membrane and participate in microspore fate change towards sporophytic pattern and may be involved in stress signaling [177].

### 1.4. Stresses and How They Affect Nucleus at the DNA and Histone Levels and Gene Expression

The crosstalk between DNA methylation, histone methylation/acetylation, and genome-wide chromatin remodeling is required for microspore reprogramming, totipotency, and initiation embryogenesis. It was shown that low levels of H3K9 methylation favor embryogenesis initiation, while its increased level is needed for embryo differentiation progresses. The process of histone demethylation and its methylation correlates with the expression of *BnHKMT SUVR4-like* and *BnLSD1-like* genes. Interestingly, BIX01294 small molecule (an inhibitor of H3K9 methylation) may promote microspore reprogramming in *Brassica napus* and *Hordeum vulgare* [92]. If used at advanced stages, the same molecule impairs embryo formation. Thus, the initiation of embryogenesis requires low H3K9 methylation while embryo differentiation its high levels [68]. Moreover, demethylation of histone H3K27me3 is critical for acquisition of callus formation (see review by Pasternak and Dudits [178]). The other histone modification, namely the acetylation of lysine residues within the N-terminal tail of histones H3 and H4 (associated with actively transcribed genes and open chromatin state), promotes microspore embryogenesis. The increased histone acetylation promotes microspore reprogramming and cell totipotency [179]. For sure, such multilevel machinery being unbalanced may affect genome functioning and induction of variation under in vitro tissue culture conditions.

### 1.5. Chromosome Doubling

The development of a diploid plant from microspores requires a chromosome doubling stage. The process may proceed spontaneously or may be induced artificially by chromosome segregation blocking mitotic inhibitors such as colchicine [180]. In cereals, the haploid genome undergoes spontaneous diploidization with the frequency ranging from 40 to 50% in *Brassica napus* and 90% in barley and rye [102,181,182,183]. Chromosome spontaneous doubling mechanism assumes nuclear fusion in cereal species [184]. It is considered a preferred way of obtaining double haploid plants as this mechanism avoids induction of mutagenesis, usually associated with artificial chromosome doubling. Artificial chromosome doubling may involve colchicine, a poisonous and mutagenic alkaloid compound extracted from bulbs and seeds of autumn crocus or meadow saffron (*Colchicum autumnale*). Its action relies on preventing the microtubule formation and chromosome segregation resulting in chromosome doubling [180]. The process may induce large chromosomal rearrangements [185,186] resulting in somaclonal variation.

### 1.6. Transcriptome Changes

Many studies have shown changes in the plant transcriptome under the influence of stress factors [187,188,189]. Plant regeneration via anther culture requires the application of several stresses, including cold treatment [190]. The stress treatment allows induction of embryogenesis in the haploid uninucleate microspore [191]. Under such conditions, haploid microspores need to switch their gametophytic fate [192]. In barley before and after applying stress, significant transcriptome changes (3382 genes’ expression changes) are indicated by RNA-seq analysis. Among genes with altered expression, glutathione S-transferases (check for Section 1.8 Biochemical Aspects), heat shock proteins, and ribosomal subunit proteins were indicated. Moreover, activation transcription factors in early embryogenesis, hormone biosynthesis, hormonal signaling, and genes involved in secondary metabolism were shown [193]. Interestingly, differential expression of genes involved in plastid transcription, translation, and starch synthesis was observed in barley capable of regenerating preferentially green plants vs. that regenerating albino plants. The expression of granule-bound starch synthase I (*GBSSI*) gene in early-mid microspores correlated with a genotype ability to regenerate green plants via androgenesis [194]. Results concerning the role of nuclear and plastic-encoded polymerases in albino and green plant regeneration indicating antero- and retrograde phenomenon in barley were presented from the same group [195]. Research conducted on *Arabidopsis* *thaliana* has shown that the transcriptome composition changes significantly during callus induction in plant tissue culture, proving the high dynamics of DNA methylation [196]. It has been found that hypomethylation is characteristic of active transcription, while transcriptional repression is associated with increased DNA methylation level resulting from changes in the chromatin structure [197,198]. Protein transcriptional regulators mediate the regulation of transcription. Transcription regulators alter nucleosome structure via acetylation, deacetylation, or ATP-dependent nucleosome structure changes [199]. Among regulators studied by now, the VIP1 protein present in the nucleus [197] exhibited overexpression in tobacco cells inhibiting shoot formation, not affecting callus development and root formation. In turn, the work of Pischke et al. [200] knowledge on transcriptome alteration in the synthesis of cytokinins, compounds necessary for the proper growth and development of plants. Cytokinins participate in signaling in callus tissues [201,202,203,204]. The other particles that exhibited a change in the expression levels are small RNAs (namely, a unique microRNA set). Some of these particles are being highly expressed, influencing multicopper oxidases (laccases). When suppressed, laccases prevent lignification and cell wall thickening, keeping the cells in an undifferentiated, meristematic state. The microRNAs expression correlated with the transition from the undifferentiated to the differentiated stage of cells [205]. The study demonstrates that copper ions may influence the cell’s biochemical pathways but in complexes that may affect epigenetic machinery (see review [206]). Recent studies in barley have also demonstrated that different micro RNAs are involved during microspore development. Some of them appear due to stress responses. Furthermore, miRNA-directed regulation of several transcription factor families (ARF, SPL, GRF, and HD-ZIPIII) was needed for the transition of microspores toward the embryogenesis pathway [207]. Differential expression of small RNAs was also denoted in the early development of broccoli (*Brassica oleracea* var. *italica*) pollen [208]. Differences in expression of some miRNAs were shown depending on the developmental stages of broccoli pollen. They were involved in the developmental transition from uni- to bi- and trinucleate pollen grains. Some of the miRNAs were specific for the phases. Based on gene ontology functional annotation, the miRNAs targeted plant organ formation, morphogenesis, and male reproduction development [209].

### 1.7. Retrograde and Anterograde Signaling

**Figure 1:**
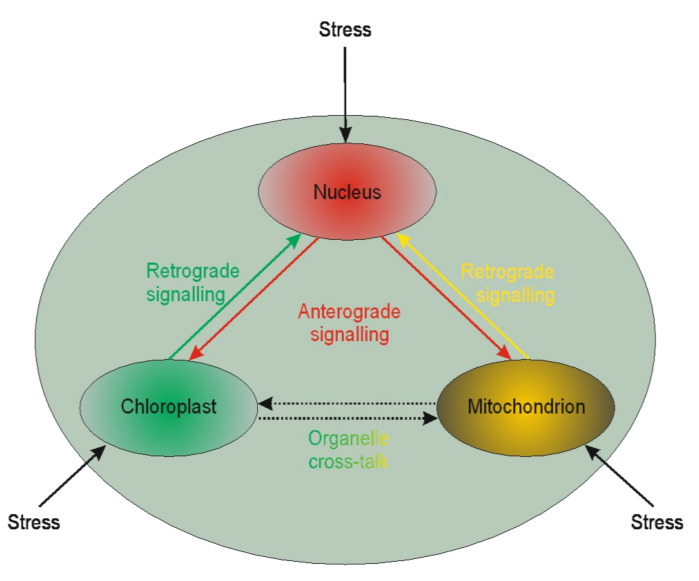
Schematic illustration of interplay between cellular organelles. Anterograde is the signaling from the nucleus towards chloroplasts or mitochondria, whereas the reversed direction is called retrograde signaling. The communication between mitochondria and chloroplast (and other organelles except but nucleus) is called cross-talk.

It is becoming evident that nuclear genes’ transcription regulation may be modulated by plastid gene expression [210,211,212]. Such regulation is called retrograde signaling. The reversed interaction when nucleus genes influence plastids’ genome functioning is called anterograde [213] (Figure 1).

Little is known of the two interactions in the context of microspores. The available literature is devoted chiefly to chloroplasts. Based on chloroplast biogenesis, retrograde signaling could be divided into “biogenic” and “operational” signals. The “biogenic signals” are the signals exhibited during early chloroplast development, whereas the “operational signals” are related to chloroplasts’ normal function in mature plants. Based on the signals’ origin, the retrograde signaling pathways in chloroplasts may include tetrapyrroles biosynthesis, redox state, chloroplast gene expression, reactive oxygen species, and protein import into plastids. The retrograde signaling pathway was described in barley mutant *albostrians*, which lacks plastid ribosomes and shows reduced amounts and/or activities of nuclear-encoded plastid proteins (the small subunit of ribulose-1,5-bisphosphate carboxylase⁄oxygenase (Rubisco), ferredoxin NADP+ reductase, and enzymes of the Calvin cycle) [214]. Inhibition of tetrapyrrole biosynthesis and plastid gene expression activity resulted in the moderated expression of nuclear starch biosynthesis genes in tobacco [215]. It is well known that in vitro plant regeneration involves stressful conditions that induce chloroplast proteome remodeling. Nuclear factors regulate chloroplast gene expression, and this form of anterograde regulation has roles in plant adaptation to abiotic stress [216]. Furthermore, retrograde and anterograde signaling was demonstrated in barley in the case of albino plant regeneration via androgenesis [193,194]. The other line of evidence suggests that retrograde mitochondrial signaling may also be essential for plants. For instance, mitochondria have been proposed to play a vital role in programmed cell death (PCD) in anther tapetum cells. PCD is crucial during anther development providing lipids coating pollen exines [217]. The role of PCD in the tapetum was also demonstrated in sunflower [218]. However, it could not be excluded that such similar signaling may act for anther cultures. Thus, the retro and anterograde signaling pathways may affect organelle functioning’s biochemical level, linked to epigenetic aspects of the in vitro tissue culture-induced variation.

### 1.8. Biochemical Aspects

**Figure 2:**
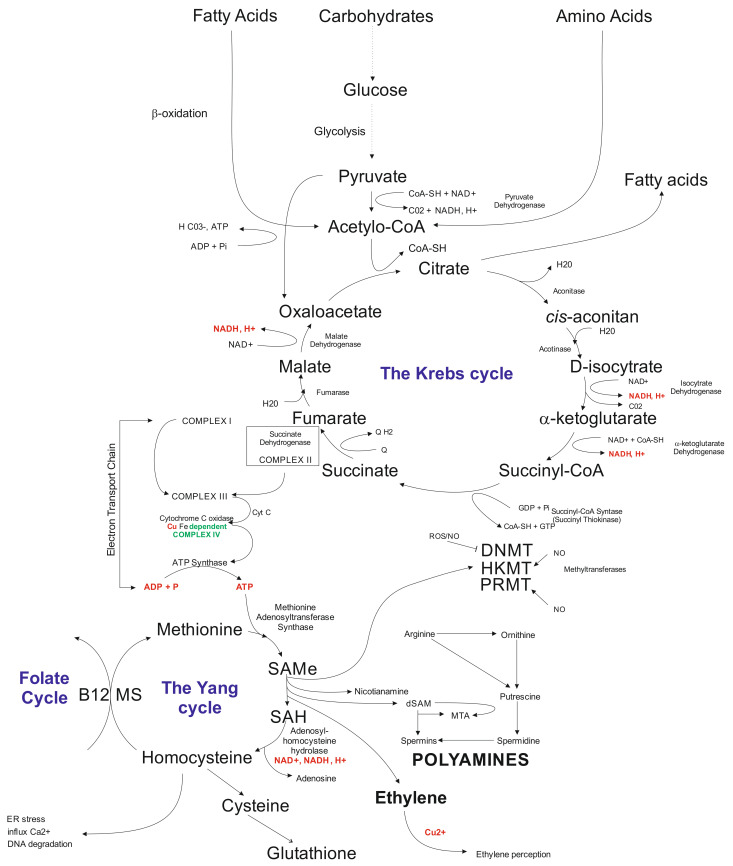
Schematic illustration of biochemical cycles that may participate in the induction of in vitro tissue culture variation. Briefly, under cold treatment and darkness and maltose presence, carbon starvation stress results in disfunction of glycolysis affecting the Krebs cycle. Disbalance in the Krebs cycle could be sensed at the electron transfer chain level via complex II and problems with ATP synthesis, which influence the Yang cycle. The latter is responsible for producing SAM involved in 80% of the cell compounds’ methylation processes, including genomic cytosines. The Yang cycle’s mall functioning may also influence glutathione production (antioxidant) used but the cell during oxidative stress. Oxidative stress may modify methylated cytosine inducing point mutations. Furthermore, byproducts of the Yang cycle may regulate transcriptome as the result of abiotic stresses. Abbreviations: B12 (vitamin B12); CoA (Coenzyme A (acyl-CoA); CoASH (coenzyme A not attached to acyl group); DNMT (DNA methyltransferase); dSAM (decarboxylated SAM); ER stress (endoplasmic reticulum stress); HKMT (histone lysine methyltransferase); PRMT; MS (methionine synthesis); MTA (5′-methyl thioadenosine); NAD (nicotinamide adenine dinucleotide); NADH (1,4-dihydro-nicotinamide adenine dinucleotide); PRMT (protein arginine N-methyltransferase); ROS (radical oxygen species); SAMe (S-adenosyl-L-methionine); SAH (S-adenosylhomocysteine).

Another layer that is being affected by in vitro tissue culture plant regeneration relates to biochemical pathways. The available data indicate that callose present in the subintinal layer [128] of microspores may affect the Krebs’ cycle via complex II involved in electron transport chain (ETC) [62]. Problems with ATP synthesis may disturb the Yang cycle [219], followed by DNA methylation problems [220] and induction of mutations [221], and possibly activation of transposable elements (TEs) [222]. Indirectly, the Yang cycle is responsible for spermine and spermidine synthesis [223]. The compounds may participate in gene expression regulation [224]. Furthermore, the Yang cycle is essential for the cell’s glutathione synthesis as an antioxidant reagent preventing modification of, for example, methylated cytosines. It was documented that glutathione significantly improved plant regeneration via anther culture in rye [102]. Possibly, that disturbances in the Krebs’ cycle may change fatty acid synthesis (Figure 2). Fatty acids may influence, for example, gene transcription [225] and glycolysis. Furthermore, glycolysis could be disturbed under carbon starvation stress [226]. Thus, in vitro tissue culture plant regeneration is a complex, multidimensional process affecting all cell functioning levels. Understanding the relationships among all of the system’s components is vital for the elaboration of knowledge-based approaches of in vitro plant regeneration and regulation of the levels of somaclonal variation.

## 2. Conclusions

The available literature demonstrates that TCIV is a complex phenomenon, including the mucilage layer, the cell wall, the cell membrane, and the interactions of cellular organelles. The other layer involves transcriptome functioning changes. Furthermore, all these alterations may be linked to biochemical pathways responsible for DNA methylation and sequence variation.

The crucial factors influencing the balance between the different aspects of cell organization are abiotic stresses affecting in vitro tissue cultures and pretreatment stages.

The establishment of all the linkages involved in the TCIV requires further interdisciplinary studies that would extend our knowledge of cell functioning under in vitro tissue culture conditions. Thus, the presented review was an attempt to gather the scattered data in a single place.

## Figures and Tables

**Table 1:** The arrangement of molecular techniques used to study TCIV.

Technique	Description	Pros	Cons
AFLP [27]	A combination of the RFLP and RAPD techniques. It involves DNA digestion with two endonucleases (usually four and six cutters) for genome reduction purposes. Adaptors complementary to sticky ends are ligated, and such products are amplified to enhance the signal. Finally, selective amplification with primers having three selective bases at 3′-ends further reduces genome complexity. The amplification products are visualized on X-rye films.	Fast, highly reproducible and adaptable to many variants.	Requires electrophoretic equipment, markers are not assigned to chromosomes.
Bisulfite-based sequencing [65]	A method for DNA methylation analysis based on converting genomic DNA by using sodium bisulfite.	It is qualitative, quantitative and efficient approach to identify 5-methylcytosine at single base-pair resolution.	It cannot discriminate between 5-methylcytosine and 5-hydroxymethylcytosine. It only converts single-stranded DNA (ssDNA). Costly.
DArTseqMet [66]	Uses the hybridization of DNA with probes placed on microarrays. The source of variation in DArT is single nucleotide polymorphism in the restriction site region, insertion deletion, changes in DNA methylation patterns, and repeated sequences.	Permitted for identifying a large number of markers that could be employed in quantification procedures. Does not require knowledge of the DNA sequence.	It is necessary to develop genomic libraries to obtain probes. The dominant nature of DArT markers.
metAFLP [46]	It is a variant of the AFLP. However, two AFLP platforms are used simultaneously, exploiting the properties of Acc65I and KpnI isoschizomers. Acc65I is sensitive towards site DNA methylation, whereas KpnI is not.	As for AFLP. It allows the identification of markers reflecting sequence variation and DNA methylation changes. Could be used to quantify variation.	As for AFLP.
Methylseq [42]	An NGS variant of the bisulfite-based sequencing approach. It is used to study different types of genomic DNA methylation.	Measuring the DNA methylation status of a very large number of regions where DNA sequence is known. Sensitive, highly specific with very low background, reproducible, and simple to execute. It is relatively inexpensive, requiring fewer reads on next-gen sequencers.	Methyl-seq assays only the CpGs in a specific subset of HpaII restriction enzyme cleavage sites. This creates a problem measuring methylation quantitatively.
MethylRAD [41]	NGS variant of the bisulfite-based sequencing approach. It could be used to study genomic DNA methylation. Instead, it uses Mrr-like enzymes to collect 32-bp methylated DNA fragments from the whole genome for high-throughput sequencing.	It allows for de novo methylation analysis using low DNA input. Delivers many markers that in some species might be mapped.	Its application requires at least 10X coverage. Costly.
MSAP [28]	The semi-quantitative MSAP approach is similar to AFLP. Utilizes the properties of the HpaII and MspI isoschizomer that differ in sensitivity towards site DNA methylation.	It allows for quantifying DNA methylation changes, suggested to overcome the limitation.	As for AFLP. Only some methylation changes could be detected. Calculation of quantitative changes usually varies from study to study.
RAPD [17]	The approach is designed to amplify DNA fragments with random, usually 10-mer primer. Separation on agarose gel is needed.	Easy to run, low-cost, many markers can be generated.	Sensitive to PCR conditions. Reproducibility problems. In its basic form may identify only sequence variation. Markers not assigned to chromosomes.
RFLP [16]	It relies on DNA digestion with endonucleases, fragment separation on gels.	Easy to run.	Time-consuming, limited number of markers can be generated.

**Table 2:** Summation of stresses that efficiently switch the developmental fate of microspores.

Abiotic Stress	Factor	Plant
Starvation/Heat	Sucrose-free medium, 25 °C, 33 °C	Wheat, tobacco [69]
Osmotic	Mannitol solutions	Barley [70]
Heat	41 °C	Rapeseed [71]
Cold	4 °C	Pepper [72]
Cold/Heat	4 °C/32 °C	Rye [73]

## Abbreviations

3-MA	3-methyladenine
AFLP	amplified fragment length polymorphism
ATR-FTIR	attenuated total reflectance Fourier transform infrared
B12	vitamin B12
CESA	cellulose synthase
CoA	coenzyme A (acyl-CoA)
CoASH	coenzyme A not attached to acyl group
CSC	cellulose synthase complex
*cwm1*	*cell wall maintainer1*
*cwm2*	*cell wall maintainer2*
DAMPs	damage-associated molecular pattern
DArTseqMet	diversity arrays technology methylation analysis
DNMT	DNA methyltransferase
dSAM	decarboxylated SAM
ER stress	endoplasmic reticulum stress
ET	endogenous ethylene
ETC	electron transport chain ETC
exDNA	extracellular DNA
*GBSSI*	granule-bound starch synthase I
Glc	glucose
GTs	glycosyltransferases
HKMT	histone lysine methyl-transferase
MAPK	mitogen-activation protein kinase
metAFLP	methylation sensitive amplified fragment length polymorphism
MIK2	leucine-rich repeat receptor kinase LRR-RK male discoverer 1-interacting receptor-like kinase 2
MLGs	mixed-linkage glucans MLGs
MS	methionine synthesis
MSAP	methylation sensitive amplification polymorphism
MTA	5′-methyl thioadenosine
NAD	nicotinamide adenine dinucleotide
NADH	1,4-dihydronicotinamide adenine dinucleotide
NGS	next generation sequencing
PCD	programmed cell death
PM	plasma membrane
PRMT	protein arginine N-methyltransferase
RAPD	randomly amplified polymorphic DNA
RFLP	restriction fragment length polymorphisms
ROS	reactive oxygen species
Rubisco	ribulose-1,5-bisphosphate carboxylase⁄oxygenase (Rubisco)
SAH	S-adenosylhomocysteine
SAMe	S-adenosyl-L-methionine
SEM	structural equation modeling
SNPs	single nucleotide polymorphisms
SV	somaclonal variation
TCIV	tissue-culture-induced variation
THE1	receptor-like protein kinase THESEUS 1
